# Sakuranetin modulates estrogen receptor signaling in breast cancer cells

**DOI:** 10.1016/j.jff.2026.107451

**Published:** 2026-08-05

**Authors:** Jorge A. Belgodere, Britney Nguyen, Anna M. Hardgrove, Stephen W. Wheat, Isaac J. Ponder, Geoffroy E.R. Sanga Pema, Julianne G. Heath, William Broussard, Sophie R. Dietrich, Steven Elliott, Syreeta L. Tilghman, Muralidharan Anbalagan, Brian G. Rowan, Bridgette M. Collins-Burow, Simak Ali, Van H. Barnes, Elizabeth C. Martin, Jayalakshmi Sridhar, Stephen M. Boué, Matthew E. Burow

**Affiliations:** a Tulane Department of Medicine, Section of Hematology & Medical Oncology, Tulane University Health Science Center, New Orleans, LA 70112, USA; b Department of Biological and Agricultural Engineering, Louisiana State University and Agricultural Center, Baton Rouge, Louisiana 70803, USA; c Tulane Cancer Center, Tulane University, New Orleans, LA 70112, USA; d Oak Ridge Institute for Science and Education, U.S. Department of Energy, Oak Ridge, TN, USA; e Pharmaceutical Sciences Division, College of Pharmacy and Pharmaceutical Sciences, Florida A&M University, Tallahassee, FL 32307, USA; f Department of Structural and Cellular Biology, Tulane University School of Medicine, New Orleans, LA 70112, USA; g Department of Surgery and Cancer, Imperial College London Hammersmith Hospital Campus London, W12 0NN, UK; h Department of Chemistry, Xavier University of Louisiana, New Orleans, LA 70125, USA; i U. S. Department of Agriculture, Agricultural Research Service, Southern Regional Research Center, New Orleans, LA 70179, USA

**Keywords:** Flavonoids, Sakuranetin, Naringenin, Estrogen activity

## Abstract

Identification of natural, bioactive compounds has been of interest to the biomedical field, in particular for the treatment of cancer. Flavonoids are a family of compounds, present across many plant species, known for their polyphenolic structure that results in cellular bioactivity. The flavonoid, sakuranetin, is produced due to external stressors, through the naringenin biosynthetic pathway. Binding affinity and docking simulations confirmed sakuranetin binding to the estrogen receptor alpha (ERα) pocket in a manner similar to, but with weaker affinity than 17β-estradiol (E2). Due to sakuranetin having chemical similarities to estrogen, this study evaluated the potential of sakuranetin to act as an endocrine modulator in estrogen receptor-positive (ERα+) breast cancer cell lines. In the ERα + cell lines, MCF-7 and T-47D, sakuranetin treatment induced dose-dependent estrogenic activity (≥ 10 μM). Sakuranetin significantly increased colony formation and cell proliferation in the MCF-7 cell line. Breast cancer cell lines with constitutively active ER through an inserted mutation in the ERα (Y537S) demonstrated no significant changes in estrogenic activity or cellular proliferation following sakuranetin treatment, except at an elevated dose (50 μM). Finaly, sakuranetin treatment enhanced genes associated with ER signaling and significantly increased ERα-mediated gene (PGR and CXCL12) expression. These results support the role of sakuranetin as a natural estrogenic compound.

## 1. Introduction

Flavonoids are a family of natural plant polyphenols that are further subdivided based on the substituents on the aromatic rings. These compounds are detected in most parts of the plant and in a wide variety of vegetables, legumes, and fruits(Graf et al., 2005; Panche et al., 2016; Ullah et al., 2020). Common members include flavanones, flavonols, flavones, and isoflavones are synthesized naturally, but production of these compounds can be enhanced in response to external stimuli. Given their sourcing, levels of flavonoids in the human body are dependent on dietary patterns(Graf et al., 2005). Strong bioactivity of known flavonoids has been reported, including properties that elicit anticancer (Hoang et al., 2025; Li et al., 2018), antioxidant(Berim & Gang, 2015), anti-inflammatory(Deng et al., 2024), antiviral(Miyazawa et al., 2006), and antimicrobial(Stompor & Zarowska, 2016) effects. Among these, sakuranetin has been previously characterized as having anti-fungal activity reducing insect nutrition(Liu et al., 2023) and shown promise as a bioactive natural compound.

Sakuranetin was initially discovered in the bark of cherry trees (Asahina, 2006) but has since been found in other plant species and plant parts (leaves, resin, pulp)(Stompor, 2020). Although sakuranetin is not abundant in most plant species, it is produced in high amounts in response to physical or biological stress. For example, ultraviolet-C irradiation and treatment with CuCl2, jasmonic acid, or phytopathogens significantly increases sakuranetin concentration of rice leaves (Kodama et al., 1992),(Cho & Lee, 2015). Sakuranetin is produced through the naringenin biosynthetic pathway(Rakwal et al., 2000), in which the enzyme 7-*O*-methyltransferase is activated in response to external stress, leading to the degradation of the precursor naringenin and the subsequent production of sakuranetin(Salehi et al., 2019; Shimizu et al., 2012).

Structural similarity to natural estrogens, and the ability to interact/bind to an estrogen receptor (ER) have led flavonoids to be characterized as phytoestrogens(Cos et al., 2003). These interactions result in eliciting estrogenic or antiestrogenic responses(Kiyama, 2023) and has led researchers to investigate of phytoestrogens that are natural or found from cash crops. Additionally, scientific methods to extract, quantify, and purify naturally occurring phytoestrogens have also been of interest. A deeper understanding of phytoestrogen modulation of the ER, through selective agonists/antagonists, is clinically relevant and currently under investigation for the prevention and treatment of pathological conditions, such as cancer, metabolic and cardiovascular diseases, and inflammation(Paterni et al., 2014). Estrogenic activity has been correlated with the presence of a phenolic ring, but phytoestrogens can also have antiestrogenic properties, potentially due to binding competition with endogenous estrogens such as 17β-estradiol (E2)(Heldring et al., 2007; Kuiper et al., 1997; Kuiper et al., 1998).

Despite growing interest in natural compounds and agonist activity of the ER by sakuranetin precursors, notably naringenin, the estrogenic effects of sakuranetin have not been determined(Jeong et al., 2018). This study aims to evaluate sakuranetin as an estrogenic and/or antiestrogenic flavonoid with the following objectives: (1) to compare ERα binding affinity to synthetic E2 through model docking with estrogen receptor α; (2) to assess dose-dependent estrogenic and antiestrogenic effects in breast cancer cell lines endogenously expressing the estrogen receptor (MCF-7 and T-47D); (3) to evaluate the impact of sakuranetin on estrogen-mediated cell proliferation and colony formation; and (4) to examine the impact of sakuranetin on the cellular transcriptome (RNA sequencing and PCR).

## 2. Materials and methods

### 2.1. Materials

Solvents used for the study were HPLC grade methanol, acetonitrile, and water all purchased from Fisher Scientific (Pittsburgh, PA). Sakuranetin, 17β-estradiol (E2), fulvestrant (ICI 182, 780), and dimethyl sulfoxide (DMSO) were purchased from Sigma Chemical Company (St Louis, MO).

### 2.2. Cell culture

The MCF-7 cell line used for colony assays and gene expression analysis was originally obtained from ATCC. CRISPR-Cas9 engineered MCF-7 (Y537S) and MCF-7-wild type (WT) control cells were generated as previously described (Harrod et al., 2022) and used for proliferation and binding affinity assays. The CRISPR engineered T-47D (Y537S) and WT control T-47D cell lines were generously donated by Steffi Oesterreich and generated as previously described (Bahreini et al., 2017). T-47D, MCF-7, MCF-7-Y537S, and T-47D-Y537S breast cancer cell lines were maintained in in Dulbecco’s Modified Eagle’s Medium (DMEM; Invitrogen, Carlsbad, CA) with 10% fetal bovine serum (FBS, Life Technologies, Inc., Gaithersburg, MD), 1% antimycotic-antibiotic (10,000 U/mL penicillin G sodium, 10,000 μg/mL streptomycin sulphate, 25 μg/mL amphotericin B as Fungizone^®^, Invitrogen, Carlsbad, CA), 1% MEM non-essential amino acids (100×, Invitrogen, Carlsbad, CA), 1% sodium pyruvate (100×, Invitrogen, Carlsbad, CA), 1% basic minimum MEM essential (50×, Invitrogen, Carlsbad, CA), and insulin (4 mg/mL, Invitrogen, Carlsbad, CA). Media designated here as “stripped media” was used to culture cells under conditions free of exogenous estrogens and was composed of phenol red-free DMEM (Gibco, Billings, MT), 5% dextran-coated charcoal-treated FBS (DCC, HyClone, Logan, UT), 1% penicillin streptomycin (Gibco, Billings, MT), 1% basic minimum MEM essential and MEM non-essential amino acids, 1% sodium pyruvate, and 1% Glutamax (100×, Gibco, Billings, MT). All cells were cultured in a humidified tissue culture incubator, maintained at 37 °C and 5% CO_2_.

### 2.3. ER binding assay

Following the manufacturer’s protocol, sakuranetin-ER binding affinity was evaluated using the ThermoFisher LanthaScreen binding assay (Waltham, MA). Briefly, stock solutions were prepared (sakuranetin, DMSO (negative control), and E2 (positive control)), serially diluted, and 10 μL of each solution were pipetted into the assay plate. Fluormone^™^ ES2 Green tracer (5 μL), ER Alpha-LBD (GST,5 μL), and terbium anti-GST antibody (5 μL) solutions were added, and the plate was rocked on a shaker incubator, protected from light, at room temperature. Fluorescence was analyzed at 495/520 nm using an Agilent Biotek Cytation5 and IC_50_ values were calculated. Reported data represents the mean ± SEM of two independent experiments.

### 2.4. Molecular docking and modeling

Molecular docking and modeling were performed following previously published studies(Belgodere et al., 2024; Belgodere et al., 2025). Briefly, 3D-structures were downloaded from the RCSB Protein Data Bank (RCSB PDB) website and Molecular Operating Environment (MOE) modeling software was used for docking studies. Fig. S1 presents the compounds used as ligands for docking studies, built in MOE. Required hydrogens were added using the MMFF94 force field (0.1 RMS kcal/mol/A^Λ^2). The WT ER-E2 ligand complex was characterized by a hydrophobic cavity created by 12 -helices and was arranged in three layers, with the binding cavity (H3, H8, H11, and H12) in the lower half of the domain. Within the WT binding pocket, the E2 phenolic hydroxyl group (A ring) made indirect hydrogen bonds, through a water molecule, with the carboxylate of Glu353 and guanidinium group of Arg394. The secondary alcohol on the cyclopentane (D ring) formed a hydrogen bond to the sidechain imidazole nitrogen of His524 on H11. The AF-2 (Y537S)-E2 ligand complex was characterized similarly, with the binding cavity (H3, H4, and H12) in the lower half of the domain. Y537S-E2 binding resulted in the phenolic hydroxyl (A ring) forming a hydrogen bond to the carbonyl of Glu353 and the secondary alcohol (D ring) formed a hydrogen bond to the sidechain imidazole nitrogen of His524. Ligands were subjected to conformational search application in the LowModeMD and the ‘Dock’ module and alpha triangle matcher were used for the placement of ligands in the binding pocket. Poses were generated by aligning ligand triplets of atoms on triplets of alpha spheres. A rescoring of binding pose was completed through the GBVI/WSA ΔG, which estimated the free energy of binding of the ligand-pose interaction. The top 5 poses with the top ‘S’ scores were evaluated through visual inspection of the docked complexes. The ligand pose that satisfied the visual inspection and the lowest ‘S’ score, was taken as final binding pose.

### 2.5. ERE-luciferase assay

MCF-7 and T-47D cell lines were grown in stripped media for 24 h, lifted and plated (5 × 10^5^ cells/well) in a 24-well plate (Corning, Corning, NY) and cultured for 24 h. Following the manufacturer’s protocol, cell transfection used 0.1 μg ER(2)-luc plasmid (Panomics, Fremont, CA) and Effectene transfection reagent (Qiagen, Hilden, Germany). After 6 h, cells were treated with DMSO, E2 (0.1 nM), ICI 182, 780 (1 μM), sakuranetin (0.1–25 μM), and E2 (100 μM) + sakuranetin (0.1–25 μM). After 18 h, media was removed and luciferase activity was determined through Luciferase Assay Substrate (Biotium, Fremont, CA) and read using a Lumat tube luminometer (Bertholdt, Bad Wildbad, Germany) as previously described(Belgodere et al., 2024; Belgodere et al., 2025). Reported data represents mean ± SEM of three independent experiments.

### 2.6. Colony formation assay

The MCF-7 cell line was plated (1000 cells/well) in stripped media in 12-well culture dishes (VWR, Radnor, PA). After 24 h, cells were pretreated with either vehicle (DMSO) or 100 nM ICI 182780. After 4 h, vehicle (DMSO), 10 μM sakuranetin, 100 nM ICI 182, 780, or 0.1 nM E2 were added directly to the wells. At endpoint (10 days), cells were fixed with 25% glutaraldehyde (Fisher Scientific, Hampton, NH) for 15 min, stained with 0.1% crystal violet (Sigma-Aldrich, St. Louis, MO) in 1% methanol (Fisher Scientific, Hampton, NH) for 15 min, rinsed with PBS, and allowed to dry. QuPath(Bankhead et al., 2017) (Version 0.70) was used to image and analyze colonies. Data represents the mean colony count ± SEM of three independent experiments. Positive colony stain was determined by colonies greater than 50 pixels, or ~ 30 cells/colony.

### 2.7. Western blot

MCF-7 cells were grown in charcoal stripped media (5% charcoal-dextran stripped FBS) phenol red free DMEM for 24 h. Once confluent, cells were transferred and cultured in 100-mm dishes for 24 h, followed by treatment with DMSO, E2 (0.1 nM) or sakuranetin (50 μM). treatment with TNF-α (50 nM) was used as control for induction of cell death. After 48 h, cells were pelleted then lysed with 100 μL of mammalian protein extraction reagent (MPER) supplemented with 1% protease inhibitor and 1% phosphatase inhibitor (Thermo Scientific, Waltham, MA, USA), incubated on ice for 10–20 min, then centrifuged at 14,800 RPM for 10 min at 4 °C. The supernatant was evaluated for relative protein concentration using a NanoDrop Ultra Spectrophotometer (Thermo Fisher Scientific, Waltham, MA, USA). Proteins were heat-denatured at 100 °C, and max protein was loaded per lane on NuPAGE 4–12% Bis-Tris Gel (Thermo Fisher Scientific, Waltham, MA, USA). Protein was then transferred to nitrocellulose membranes using the iBlot 3 Western blot transfer system per manufacturer’s instructions (Thermo Fisher Scientific, Waltham, MA, USA). Membranes were incubated in 1% Tris-Buffered Saline with Tween 20 (TBS-T) (BioLegend, San Diego, CA USA) with 5% Bovine Serum Albumin (BSA, Fischer Bioreagents, Waltham, MA, USA) for 1 h followed by 4 °C incubation overnight with primary antibodies diluted 1:2000 for loading control (β-Actin: BioRad, Hercules, CA USA, Catalog Number 12004163) and 1:1000 for PARP1 (Cell Signaling Technology, Danvers, MA USA, Catalog Number 9532). After three 5 min washes in 1% TBS-T, membranes were incubated with appropriate secondary antibodies diluted 1:10,000 in 5% BSA and 1% TBS-T blocking buffer for 1 h at room temperature (IRDye 680RD, Catalog Number 926–68072) (LiCor Biosciences, Lincoln, NE, USA). Following incubation with secondary antibodies, membranes were washed three times for 5 min in 1% TBS-T, and blots were analyzed by the ChemiDoc MP (BioRad, Hercules, CA, USA). Band density was quantified through ImageJ version 1.54. Relative protein density was normalized to β-Actin as loading control. Data represent the mean value ± SEM of three independent experiments, normalized to vehicle (DMSO) group as control (set to 1). Full blots are shown available in Supplemental Fig. S2. One-way ANOVA, Dunnett’s multiple comparisons test was used for statistical analysis, compared to DMSO.

### 2.8. Proliferation assay

Wild type (WT) MCF-7 and T-47D and corresponding ER mutant (MCF7-Y537S and T-47D-Y537S) cell lines were grown in stripped media for 48 h. After 48 h, cells were plated (2000 cells/well) in 96 well plates, incubated for 24 h, and then treated with DMSO, E2 (0.1 nM), ICI 182, 780 (1 μM), or sakuranetin (10, 25, and 50 μM). After 5 days, cells were fixed with glutaraldehyde, stained with crystal violet, eluted in 33% acetic acid (Sigma-Aldrich, St. Louis, MO), and absorbance was analyzed at 590 nm using a plate reader (Cytation 5, Gen 5 version 3.12). Reported data represents the mean ± SEM of three independent experiments.

### 2.9. RNA-sequencing

RNA-sequencing sample preparation and data processing was performed using methods from our previous published work(Belgodere et al., 2024; Belgodere et al., 2025). Briefly, the MCF-7 cell line was cultured in stripped media for 48 h, treated for 24 h (DMSO or 10 μM sakuranetin), and then collected for total RNA extraction (Direct-zol RNA purification kit, Zymo research, Irvine, CA). RNA was sent to BGI and evaluated using a DNB-G400 (MGI Tech, San Jose, CA). Raw data were aligned to the human reference genome (HISAT), gene expression values were identified by TPM, and detected by RSEM. Unbiased pathway analysis was performed (Enrichr, MSigDB Hallmark gene set (Chen et al., 2013; Kuleshov et al., 2016; Xie et al., 2021)), top upregulated and downregulated pathways, and significantly changed genes (adjusted *p* < 0.05) were reported. Raw data files were uploaded to the NCBI’s Gene Expression Omnibus(Edgar et al., 2002) (GEO Series accession #GSE277758). Reported data represents three independent experiments and gene expression is provided in Supplemental file 1.

### 2.10. Quantitative PCR of ER-mediated genes

The MCF-7 cell line was plated (5000 cells/cm^2^) in 25 cm^2^ flasks (VWR, Radnor, PA) and cultured until 70% confluent. Once confluent, media was aspirated from flasks, flasks were washed 3 × with PBS to remove exogenous estrogens and cells were cultured in 5% stripped media for 48 h prior to treatment with E2 (100 nM), sakuranetin (10 mM), ICI 182, 780 (1 μM) or vehicle control (DMSO) for 4 h. After 4 h, additional DMSO was added to the E2-, DMSO-, and sakuranetin-treated flasks and additional E2 or sakuranetin were added to the ICI-treated flasks. Cells were collected after 24 h and collected for total RNA extraction (RNeasy Mini Kit, Qiagen, Hilden, Germany). 1 μg total RNA was reverse transcribed to cDNA (iScript cDNA SuperMix, BioRad, Hercules, CA), and quantitative real-time PCR was performed (BioRad CFX Connect Real-Time System, v4.006; BioRad, Hercules, CA and BioRad IQ SYBR green Supermix, BioRad, Hercules, CA). Gene expression was calculated following the ΔΔ(Ct) method and used β-actin as the reference gene. Primer sequences are available in Supplementary Table S1. Samples were normalized to vehicle control (DMSO) which was designated as 1. Reported data represents the mean ± SEM of three independent experiments.

### 2.11. Statistical analysis

Statistical analyses were performed using GraphPad Prism (v10.0.2, GraphPad Software, San Diego, CA). One-way ANOVA with multiple comparisons (Dunnett’s) was performed and *p*-values of <0.05 were considered significant.

## 3. Results

### 3.1. Evaluation of sakuranetin ER-α binding affinity and molecular docking simulations

**Fig. 1.**
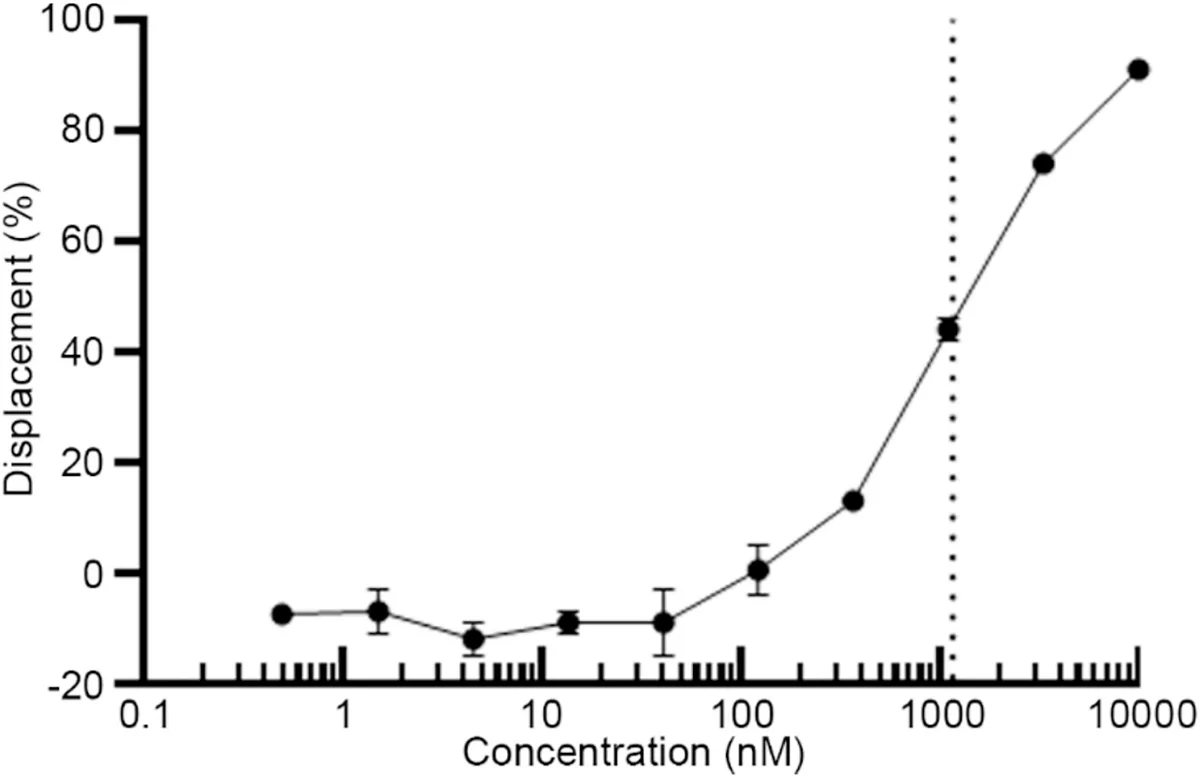
Relative binding affinity of sakuranetin to the ER pocket requires concentrations >1000 nM to initiate displacement. E2 was used as positive control, with an IC50 of 0.283 nM. Dotted line indicates calculated IC_50_ value, 1170 nM. Data represent mean ± SEM from 2 independent experiments.

The binding affinity of sakuranetin to the ER binding pocket was evaluated by generating a dose curve and evaluating the response. IC_50_ values were calculated as the concentration required to produce a > 50% displacement of the tracer was determined, and compared with the positive control, E2. Fig. 1 presents the displacement-concentration curve for sakuranetin, with the IC_50_ indicated using a vertical, dotted line. The IC_50_ for sakuranetin was 1170 nM, >4000 times greater than the positive control (E2, 0.283 nM). To better elucidate binding, we next modeled and evaluated sakuranetin-ER binding conformations.

**Fig. 2.**
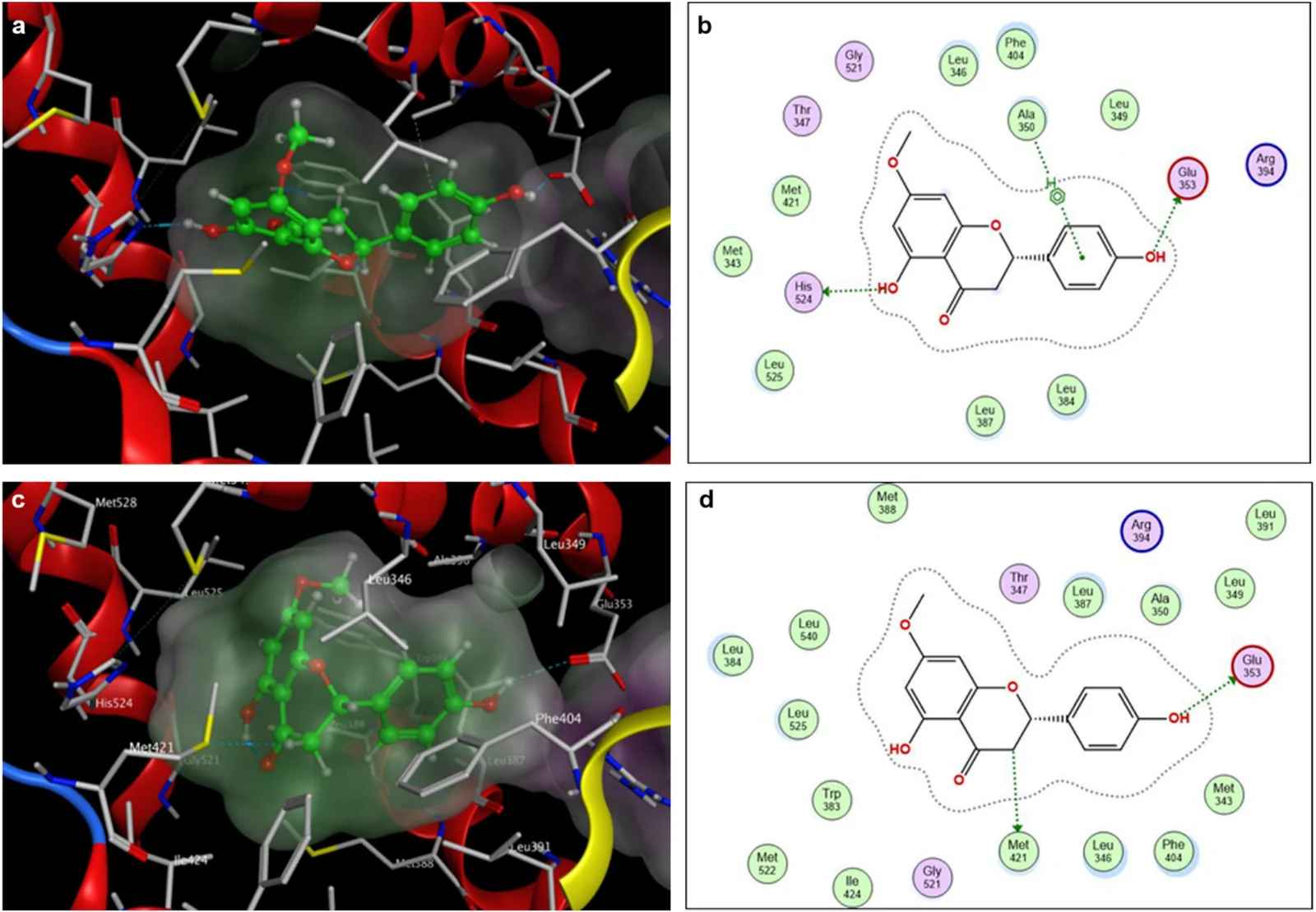
S-sakuranetin has distinct binding conformations for the WT (a-b) and Y537S mutant (c-d) ER pocket. (**a**) 3D rendering of sakuranetin binding into the WT ER-α pocket at the lowest binding score. (**b**) Ligand interaction view of sakuranetin depicting hydrogen bonds with Glu353 and His524 and a ℼ-H interaction with Ala350. (**c**) 3D rendering of sakuranetin binding into the Y537S mutant ER-α pocket at the lowest binding score. (**d**) Ligand interaction view depicting the Glu353 hydrogen bond and one with Met421.

Sakuranetin has two naturally occurring enantiomers (R & S, Fig. S1), where both oriented in the ER binding pockets similar to that of E2. For S-sakuranetin, the best binding conformations (lowest binding score of S = −6.6751 (WT ER, Fig. 2a and b) and S = −6.6962 (Y537S ER, Fig. 2c and d) formed a direct hydrogen bond with the side chain carboxyl group of Glu353, of phenolic hydroxyl group (B ring), for both WT- and Y537S-ER. The WT ER-S-sakuranetin complex formed a second hydrogen bond to His524 (A-ring) phenolic hydroxyl group (Fig. 2a and b), while the Y537S ER-ligand complex bonded the -CH_2_- (C-ring) of the chromanone to the sulfur of Met421 (Fig. 2c and d). Finally, WT ER-S-sakuranetin complex formed an aromatic ℼ-H interaction between the phenol (A-ring) and Ala350, while the Y537S ER-S-sakuranetin complex showed an overall bent structure.

**Fig. 3.**
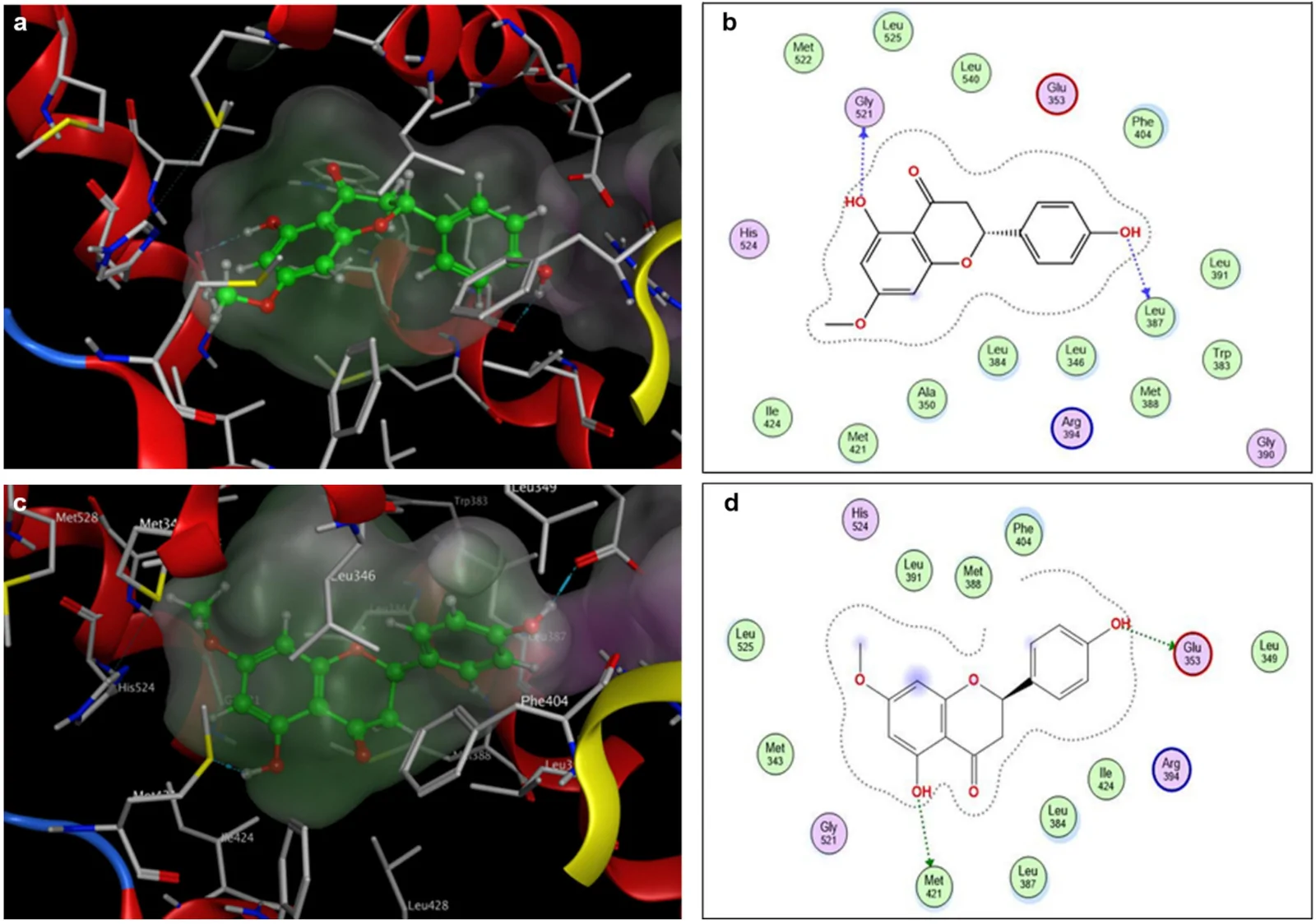
The R-enantiomer of sakuranetin binds to the ERα pocket using two distinct conformations. (**a**) 3D rendering of sakuranetin binding into the WT ER-α pocket at the lowest binding score. (**b**) Ligand interaction view of sakuranetin depicting hydrogen bonds with Leu387 and Gly521. (**c**) 3D rendering of sakuranetin binding into the y537S mutant ER-α pocket at the lowest binding score. (**d**) Ligand interaction view of sakuranetin depicting hydrogen bonds with Met421 and Glu353.

The WT-ER-R-sakuranetin binding (S = −6.5703) exhibited differences, with the bond Glu353 hydrogen bond (B ring) being replaced with the phenolic hydroxyl (B ring) and the backbone carbonyl of Leu387 and the chromanone phenolic hydroxyl group (A-ring) with the backbone carbonyl of Gly521 (Fig. 3a and b). The Y537S ER-R-sakuranetin binding (S = −6.8474) occurred with the phenolic group (B ring) hydrogen bonding to the carboxyl of Glu353, as seen for S-sakuranetin, while the chromanone phenolic group (A ring) hydrogen bonded to the sulfur of Met421 (Fig. 3c and d).

### 3.2. Sakuranetin promotes estrogenic activity in a dose-dependent manner

**Fig. 4.**
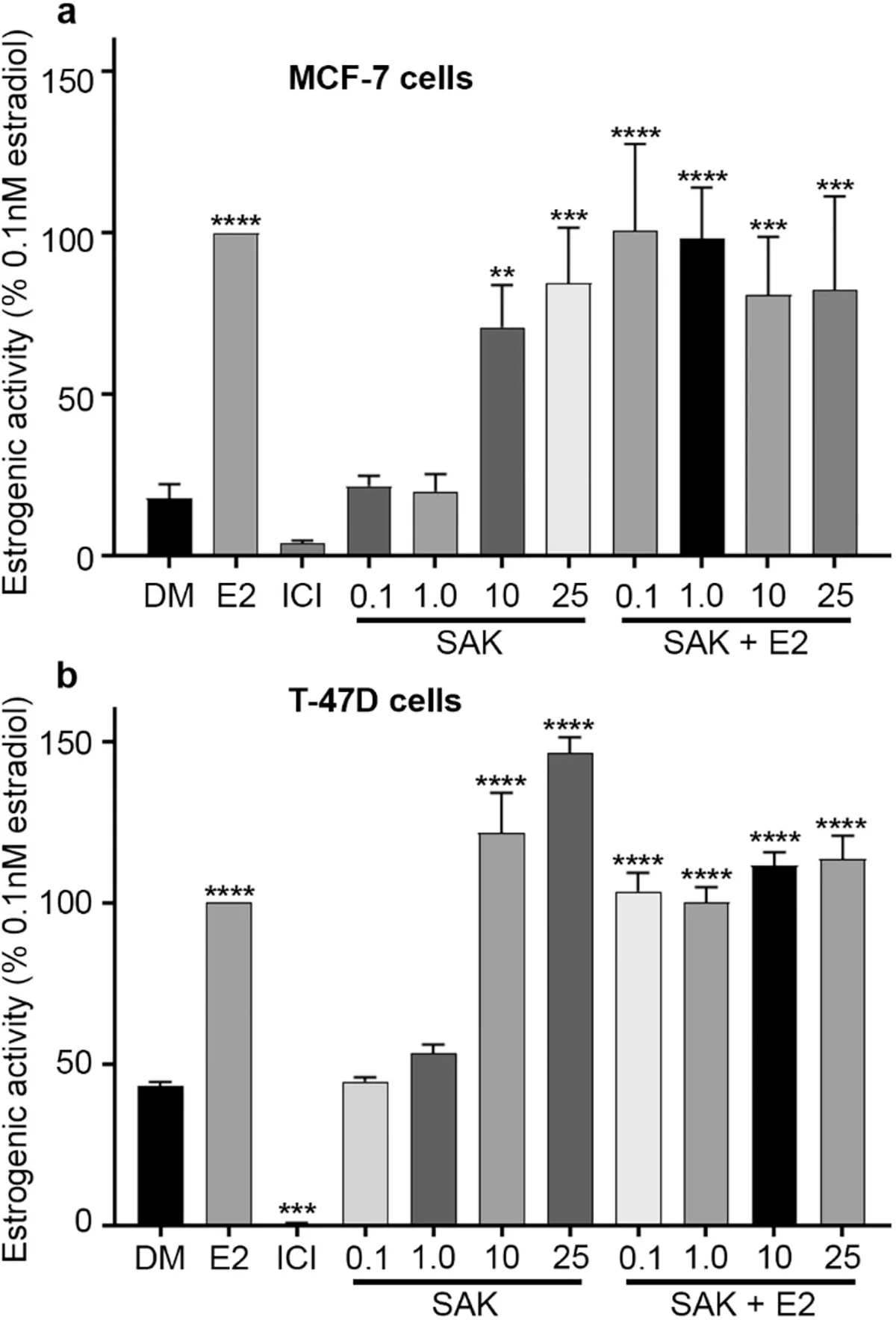
Sakuranetin elicits dose-dependent estrogenic responses in cells with endogenous ER. MCF-7 cells were transiently transfected with ER(2)-luc plasmid prior to treatment with DMSO, E2 (0.1 nM), ICI 182, 780 (1 μM), SAK (sakuranetin, 0.1, 1, 10, or 25 μM), or combinations of SAK and E2 (0.1 nM). Data represent mean ± SEM from *n* = 3 independent experiments. One-way ANOVA, Dunnett’s multiple comparisons test, compared to DMSO. **p < 0.01, ***p < 0.001, and ****p < 0.0001.

To determine the effects of sakuranetin on ER activity, MCF-7 and T-47D breast cancer cell lines were transfected with an ERE-luciferase reporter and treated with increasing concentrations of sakuranetin alone or in combination with E2 (0.1 nM). High concentrations of sakuranetin (10 and 25 μM) significantly increased estrogenic activity of MCF-7 cells compared to vehicle control (Fig. 4a) while lower concentrations of sakuranetin (0.1 and 1.0 μM) did not induce ERE-reporter activity. MCF-7 cells treated with sakuranetin, in combination with E2, had no observed additive or antagonist activity (Fig. 4a). We next used an MCF-7 cell line that had an engineered mutation in the ER (Y537S) where the resulting cells contain a constituently active ER that can be used as a novel tool to provide insight into potential selective estrogen receptor modulating activity of sakuranetin. Results demonstrated that, unlike MCF-7 cells with wild type ER (Fig. 4a), the MCF-7-Y537S cells treated with sakuranetin exhibited no significant response with increasing sakuranetin doses (1, 10, and 25 μM) or E2 (Fig. S2), compared to the DMSO control. There was however a significant decrease in activity when treated with ICI 182, 780, suggesting that sakuranetin is not an antagonist. Reporter gene activity was significantly increased in T47D cells with ≥10 μM sakuranetin addition, as observed for MCF-7 cells (Fig. 4b). As in MCF-7 cells, co-treatment of E2 and sakuranetin did not alter reporter gene activity, indicative of no additive or antagonistic effects of sakuranetin on response to E2. Similar to E2, sakuranetin co-treated with E2 had no additive or antagonistic activity compared to DMSO control (Fig. 4b). For both cell lines, sakuranetin concentrations >10 μM elicited significant changes in estrogenic activity. Therefore, we next evaluated the biological effects of sakuranetin.

### 3.3. Sakuranetin-treated cells exhibit increases in colony formation and proliferative activity

**Fig. 5.**
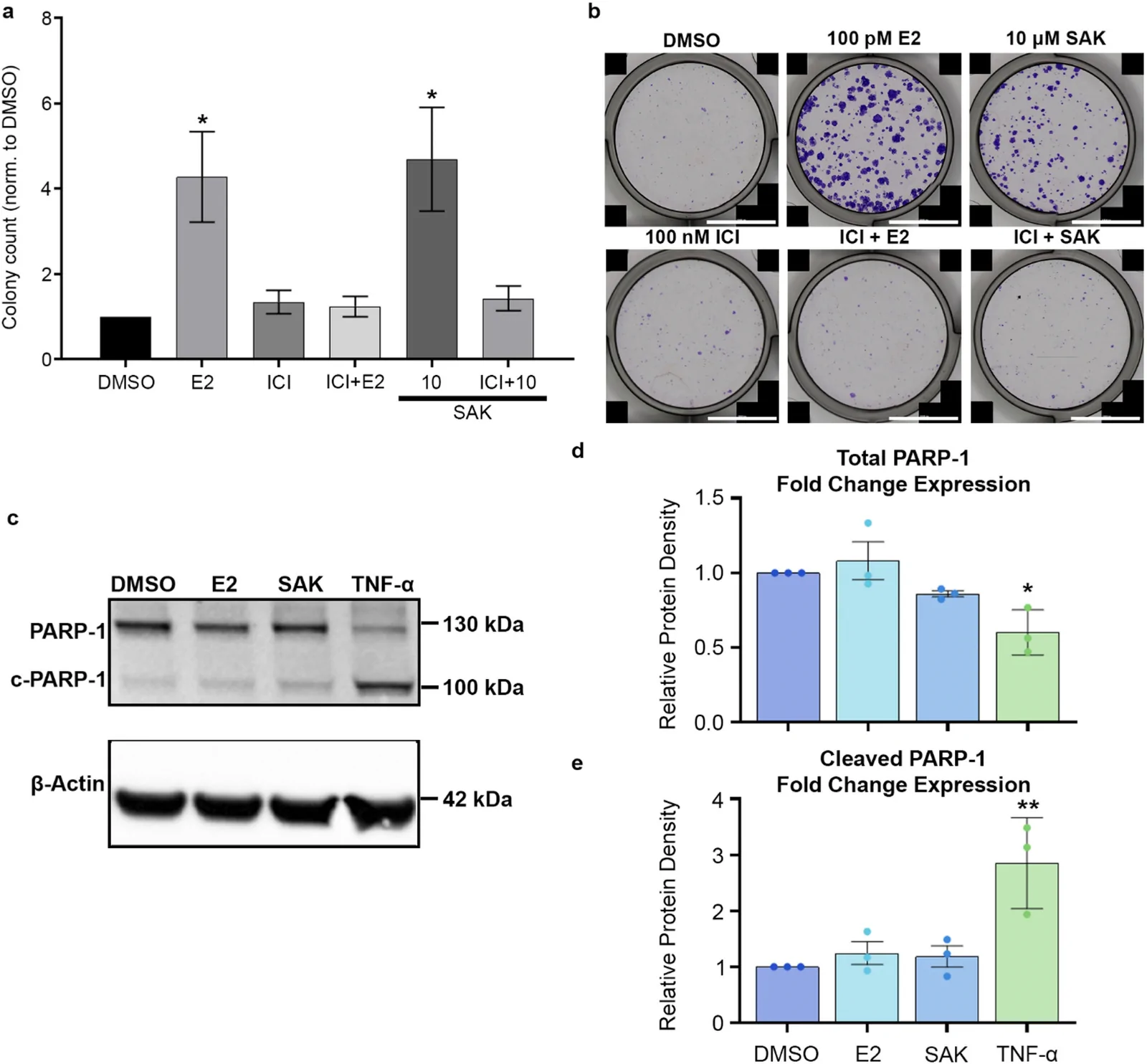
Sakuranetin significantly increases MCF-7 colony formation and does not elicit apoptosis. **a**) MCF-7 cells were treated with DMSO, E2 (0.1 nM), ICI 182, 780 (1 μM), SAK (10 μM), or combinations of sakuranetin and E2 (0.1 nM) and grown for 10 days. At each end point, cells were fixed, stained with crystal violet, and colonies were counted. Data represent mean ± SEM from *n* = 3 independent experiments. * Significantly different *p* < 0.05. **b**) Representative images of MCF-7 colony formation. Scale bars = 10 mm. **c-e**) MCF-7 cells were treated with DMSO, E2 (0.1 nM), SAK (50 μM), or positive control for cell death TNF-α (50 nM) for 48 h. Western blotting was then performed on cell lysates with representative bands shown in (c) for expression of PARP-1 (**d**) and cleaved PARP-1. (**e**) Quantified relative protein density normalized to β-Actin. Data represent the mean ± SEM of three independent experiments, normalized to vehicle (DMSO) group as control (set to 1). One-way ANOVA, Dunnett’s multiple comparisons test, compared to vehicle control (DMSO). *p < 0.05 and **p < 0.01.

The effect of sakuranetin on MCF-7 cell growth was next assessed through colony formation and cell proliferation assays. Colony formation was determined in MCF-7 cells following treatment with sakuranetin (10 μM) and cultured for 10 days. This dose was chosen based on being the lowest concentration to elicit a significant increase in estrogenic activity (Fig. 4). T-47D cells were unable to form stable and consistent colonies under serum starved culture conditions (data not shown). Treatment with sakuranetin or E2 resulted in a significant increase in colony formation, when compared to the DMSO control (Fig. 5a-b). As expected, MCF-7 cells treated with ICI did not significantly increase colony forming units, alone or in combination with either E2 or sakuranetin. Given our findings that combination treatment of E2+ SAK did not create a synergistic or antagonistic effect and yielded similar results as E2 or SAK alone (Fig. 4), combination treatments were not further evaluated. To determine if sakuranetin induces apoptosis, we treated MCF-7 cell with a high concentration of sakuranetin (50 μM) and evaluated PARP-1 protein expression (Fig. 5c-e). No significant changes were observed for total (Fig. 5c-d) and cleaved (Fig. 5c,e) PARP-1 protein expression, for sakuranetin or E2 when compared to DMSO.

**Fig. 6.**
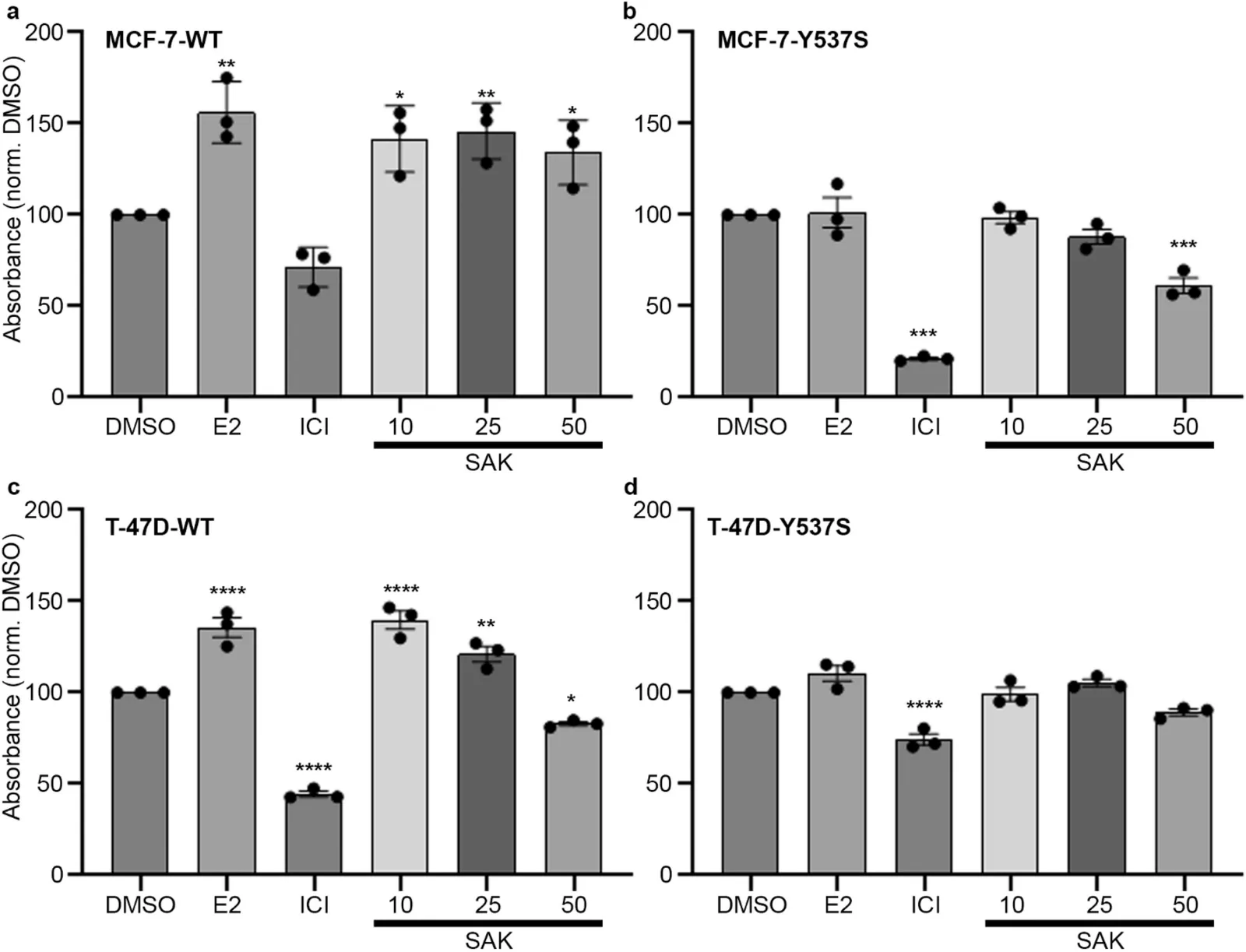
Sakuranetin promotes growth in control cell lines while largely ineffective in mutant cell lines. Cells were cultured in stripped media for 48 h, plated at 2000 cells/well in 96 well plates, and incubated for 24 h. Cells were treated with DMSO, E2 (0.1 nM), ICI 182, 780 (1 μM), and SAK (10, 25, and 50 μM). At each end point cells were fixed, stained with crystal violet, eluted, and absorbance was recorded at 590 nm. Data represent mean ± SEM for 3 independent experiments. One-way ANOVA, Dunnett’s multiple comparisons test, compared to DMSO. *p < 0.05, **p < 0.01, ***p < 0.001, and ****p < 0.0001.

To further investigate sakuranetin’s interaction with the ER and proliferation, we next evaluated the impact of sakuranetin on two ER Y537S cell lines (T-47D-Y537S, MCF-7-Y537S) and WT control lines. Our prior findings showed no estrogenic response below 10 μM (Fig. 4), therefore sakuranetin concentrations included 10, 25, and 50 μM. To determine potential antagonistic effects of sakuranetin in ER Y537S cell lines, we increased dosage to 50 μM. Similar to the MCF-7-WT cell line (Fig. 6a), sakuranetin- and E2-treatment in the T-47D-WT cell line (Fig. 6c) exhibited significant increases in cell density when compared to DMSO. The T-47D- and MCF-Y537S mutant cell lines (Fig. 6b and d), however, showed no significant changes in cell density following treatment with either E2 or escalating doses of sakuranetin (10–50 μM), when compared to DMSO. Only the MCF-7-Y537S cell line treated with 50 μM sakuranetin resulted in a significant decrease in cell density compared to DMSO (Fig. 6b). Given the changes in biological processes and responses suggesting that sakuranetin acts as an ER agonist, we next evaluated sakuranetin regulated gene expression.

### 3.4. Sakuranetin treatment induces an estrogen response in MCF-7 cells

**Fig. 7.**
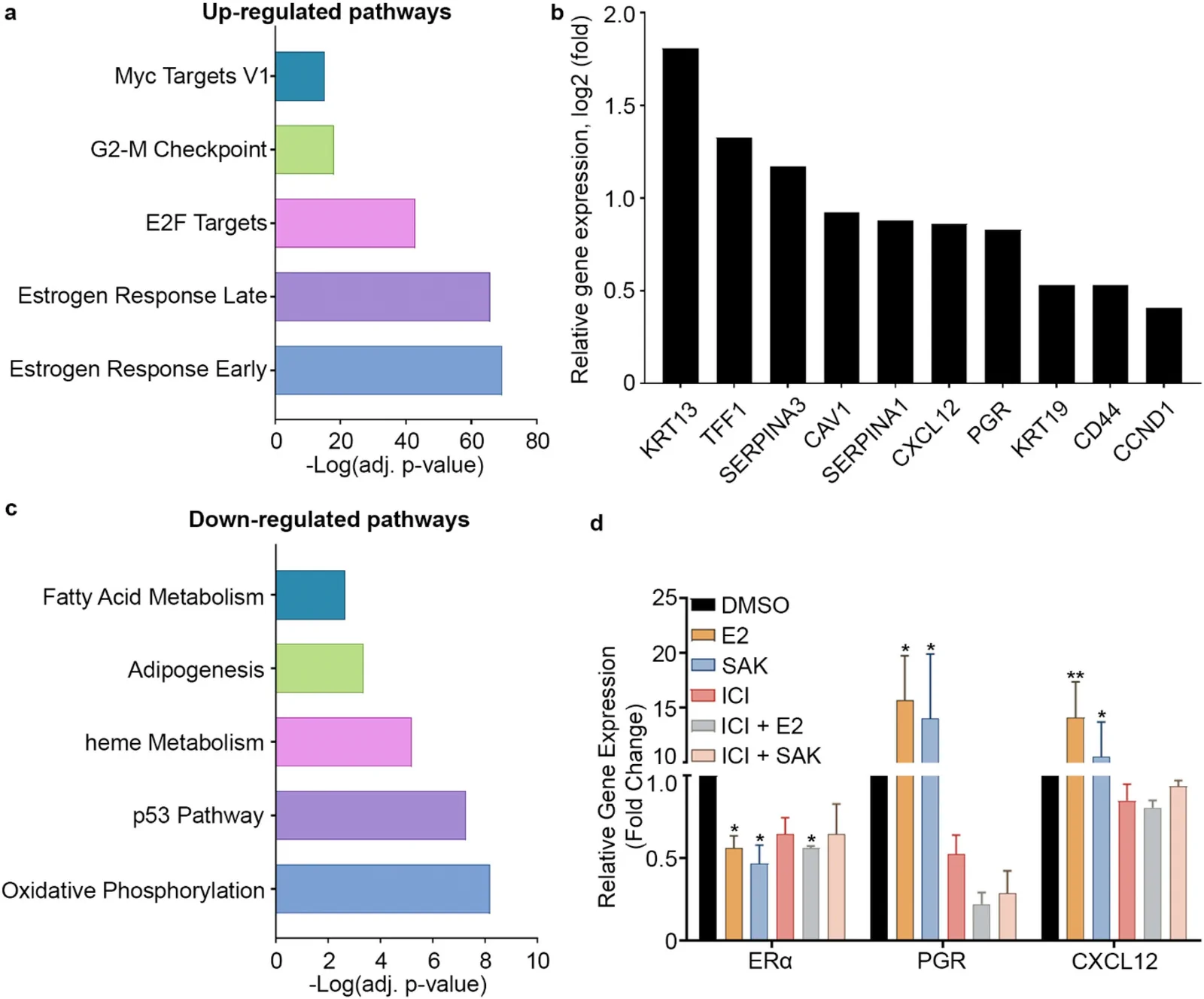
MCF-7 cells treated with sakuranetin induce significant changes within estrogen response genes and pathways. MCF-7 cells were grown in stripped media for 48 h, and then treated with SAK (10 μM, 24 h), total RNA extracted, and RNA sequencing performed. Enrichr and the MSigDB Hallmark gene set databases were used to demonstrate pathway analysis for (**a**) the top five up-regulated pathways and (**b**) relative gene expression for the relevant estrogen response genes that were significantly changed with SAK treatment. and (**c**) MCF-7 cells treated with SAK exhibit a significant change in estrogen response genes. Cells were grown in stripped media for 48 h, initially treated with DMSO, E2 (0.1 nM), SAK (10 μM), or ICI 182780 (1 μM), and then after 4 h followed with a subsequent dose of DMSO to the E2, DMSO, and SAK flasks and E2 or SAK to the ICI flasks. After 24 h, total RNA was extracted, cDNA reverse transcribed, and qRT-PCR was performed for ERα and ER response genes (PGR and CXCL12). Data was normalized to DMSO control and housekeeping gene (β-actin). **d**) The top five down-regulated pathways and relative gene expression for the relevant genes significantly down-regulated with sakuranetin treatment. RNA-seq data represent the mean from 3 independent experiments and compared to vehicle control (DMSO) and PCR data represents mean ± SEM of 3 independent experiments. One-way ANOVA, Dunnett’s multiple comparisons test, compared to DMSO. *p < 0.05 and **p < 0.01.

The global transcriptomic effects of sakuranetin were evaluated through RNA sequencing of MCF-7 cells treated with sakuranetin (10 μM) for 24 h. Findings presented in Figs. 4–6 identified 10 μM as the lowest concentration to elicit a significant response and further increases in dose did not result in a significant difference from the 10 μM treatment. The primary objective was to determine sakuranetin effect on the cellular transcriptome. Sakuranetin altered a total of 2064 genes, with 1101 genes being significantly upregulated and 963 genes being significantly downregulated (Supplemental File 1). Evaluation of significantly changed genes demonstrated the enrichment of genes associated with the Early and Late estrogen response pathways (Fig. 7a). This is further exemplified through the select increase in expression of ER transcriptional targets TFF1, SERPINA3, SERPINA1, CXCL12, PGR, KRT19, and CCND1 (Fig. 7b). Additional pathways of interest identified in our RNA sequencing, included MYC and E2F targets (Fig. 7a), two pro-proliferative signaling mechanisms. Evaluation of genes repressed by sakuranetin demonstrated enrichment of pathways associated with oxidative phosphorylation, the p53 pathway, and fatty acid metabolism (Fig. 7d). The overall greater impact on transcriptome changes elicited from sakuranetin treatment likely results from the enhanced binding affinity (Fig. 1) and docking studies (Figs. 2 and 3), compared to our prior reports for other evaluated compounds (Belgodere et al., 2024; Belgodere et al., 2025).

Given the transcriptomic signature observed following sakuranetin treatment, we next sought to determine if inhibition of the ER could reverse the observed changes in ER mediated genes through the evaluation of expression of ER, PGR, and CXCL12 with qRT-PCR. The MCF-7 cell line was pre-treated with E2 (0.1 nM), sakuranetin (10 μM), or ICI 182, 780 (1 μM) for 4 h, followed by the addition of E2 or sakuranetin to the ICI-treated cells. Mutli-combinatorial treatments with ICI 182,780 allowed for a deeper understanding of the action, or inaction, of sakuranetin-ER binding as ICI 182,780 acts as an ER degrader. At end point, gene expression was reported for MCF-7 cells and results demonstrate that MCF-7 cells treated with E2, sakuranetin, and ICI 182,780 + E2 exhibited a significant decrease in ERα expression, compared to DMSO. While PGR and CXCL12 expression was significantly increased following treatment with E2 and sakuranetin (*p* < 0.05) compared to vehicle control (DMSO) (Fig. 7d). Pre-treatment with ICI 182,780 inhibited E2 and sakuranetin induced PGR and CXCL12 gene expression (Fig. 7d), suggesting that sakuranetin is acting directly through the ER.

## 4. Discussion

Our prior reports have shown estrogenic activity for plant extracts and purified compounds eliciting significant changes in biological processes and the transcriptome of ER-α + breast cancer cell lines (Belgodere et al., 2024; Belgodere et al., 2025). These findings led to interest into the estrogenic potential of sakuranetin, a rice flavonoid, and required profiling/screening into cellular activity and changes in biological processes. Throughout the evaluation of sakuranetin, the concentrations and combinatorial treatments with E2 and ICI 182780 we carefully selected based on presented findings and were slowly optimized to reach our final working concentration (10 μM). Combinatorial treatments of sakuranetin and E2 resulted in non-significant changes and were therefore not tested in further assays.

ER-α binding (Fig. 1) for studies at the IC_50_ of sakuranetin, was similar to our previous reports for other phytoalexins, (+)-pisatin (1060 nM)(Belgodere et al., 2024) and tuberosin (2130 nM)(Belgodere et al., 2025). Although there were differences in enantiomer binding, similar binding scores indicate minimal differences in overall ER-ligand binding and therefore potential downstream effect differences would be minimized. Sakuranetin (both R and S) binding to the WT (Fig. 2a and b and Fig. 3a and b) and mutant Y537S ERs (Fig. 2c and d and Fig. 3c and d) mimicked E2 binding, in conformation and with the minimum 2 hydrogen bonds, suggesting that sakuranetin interacts with the ER and functions in an agonistic fashion, similar to E2(Carlson et al., 1997; Shanle & Xu, 2011). The modeling also showed the binding confirmation for WT ER was closed while the mutant Y537S ERs remained open, with E2 or sakuranetin. Prior work has shown an ER closed orientation promoted estrogenic behavior while an open orientation promoted anti-estrogenic behavior(Jensen & Jordan, 2003).

Binding conformation changes in the ER have previously been investigated as a potential source of drug resistance and ER constitutive activity(Fanning et al., 2016). Identifying the differences in ER-ligand complexation, through modeling, would provide insight into candidate phytocompounds that could act as novel ER modulators. While the binding and docking data confirms sakuranetin binding to the ER pocket results in non-disruptive interactions, similar to E2, additional studies were required to confirm resulting biological signaling.

Flavonoid elicited estrogenic activity has been historically well reported(Miksicek, 1993), with hormone-like activity being compared to that of natural and synthetic E2. Binding and docking data do not immediately indicate downstream signaling of critical biological actives (Moulishankar & Lakshmanan, 2020). While an important preliminary screening tool, estrogenic activity in ER-α + cell lines (MCF7 and T47D cells) was confirmed to contain a dose-dependent agonistic relationship (Fig. 4). These findings aligned with our prior findings and other published reports(Han et al., 2002; Miksicek, 1993). Additional studies are required however, to fully elucidate the mechanisms by which ER phosphorylation occurs, and to what extent.

Building from prior reports, sakuranetin was also found to significantly increase the proliferation of ER+ breast cancer cell lines (Fig. 5) further suggesting an estrogenic role for this compound in breast cancer cell lines. MCF-7 cells showed no significant change in PARP-1 protein expression, indicating no sakuranetin-induced DNA damage (total) and apoptosis (cleaved) at 50 μM (Bouchard et al., 2003). A previous study showed dose-dependent PARP-1 expression with increasing doses of apigenin, another phytocompound, in MCF-7 and MDA-MB-231 cells (Vrhovac Madunic et al., 2018). The study showed significant changes in early apoptosis occurred at 40 μM and above, indicating that phytocompounds elicit unique responses.

In addition, sakuranetin did not elicit a response in breast cancer cell lines with a constitutive active ER, supporting the mechanism of action being through the ER (Fig. 6). There was a significant decrease found at higher doses (50 μM) in the MCF-7-Y537S cells (Fig. 6b), which could indicate a complex response to sakuranetin. However, suggesting that sakuranetin may contain selective agonist/antagonist activity would require additional investigation in future experiments. Such experiments on the possible antagonistic activity of sakuranetin must be conducted to determine if the observed decrease in cell density is through ER binding, alternate signaling mechanisms, or external confounding factors. In addition, studies into clinical translation are required to determine if sakuranetin effects would also modulate ER+ tumor signaling *in vivo*.

Global trends in the ER-α + breast cancer cellular transcriptome (Fig. 7) were similar to our previously published work on other phytoalexins, (+)-pisatin(Belgodere et al., 2024) and tuberosin(Belgodere et al., 2025), when MCF-7 cells were treated under similar conditions. All three phytoalexins exhibited upregulation of ER-associated pathways. Sakuranetin (~130 genes) exhibited the highest levels of significance (−Log(adj. *p*-values)), compared to the other two (~6 genes for (+)-pisatin and ~ 60 genes for tuberosin). However, while all three exhibited upregulation of ER-associated genes, there were differences between the top downregulated pathways, specifically oxidative phosphorylation, p53, and fatty acid/adipogenesis based pathways were unique only to sakuranetin. Phytocompound induced ER-mediated gene expression has previously been reported through the treatment of other novel phytocompounds(Jiang et al., 2010; Zimmermann et al., 2010), our prior work (Belgodere et al., 2024; Belgodere et al., 2025; Boue et al., 2009), and their well-reported and established connection with ER activation through estrogen or estrogen-like binding (Hall & Korach, 2003). We further confirmed these pathways observation by evaluating traditional ER-mediated gene expression of CXCL12, PGR, and ER-α. Observed changes in the ER mediated genes were similar but varied in degree of expression increase. PGR has a strong history of being closely related to ER activity, with activation leading to intracellular downstream signaling resulting in an increase in gene or protein expression (Petz et al., 2002; Siersbaek et al., 2018). This relationship has been shown in both breast cancer cells (Bourdeau et al., 2008) and breast tumors (Creighton et al., 2009). However, the observed *in vitro* changes must be confirmed through follow up *in vivo* studies to determine if ER-mediated changes are retained.

## 5. Conclusions

Flavonoids and other phytocompounds provide opportunities to enhance commercial crops or their byproducts through increased bioactivity. Additionally, a recent shift towards natural compound research represents unique opportunities for the rice-derived compound, sakuranetin, as a natural estrogenic compound. Docking simulations showed enantiomer-specific binding to the ER binding domain, with a preference for R conformation; while ER binding affinity was lower than synthetic E2, values for sakuranetin are similar to or higher than previously evaluated phytocompounds. Docking studies also revealed that sakuranetin isomers bind to both WT and Y537S ER AF2 domains making two hydrogen bonds and without any steric clashes within the binding pocket. Initial *in vitro* screening was conducted using ER+ cells to determine the potential clinical relevance of sakuranetin. ER+ cell lines treated with sakuranetin exhibited a dose-dependent response in estrogenic activity, proliferation, and colony formation. Constitutively active ER mutant cell lines (Y537S) largely did not demonstrate a significant response to sakuranetin, suggesting that sakuranetin does not inhibit ER Y537S activity. Sakuranetin significantly increased the ER transcriptome and ER-mediated gene expression for MCF-7 cells, further emphasizing its potential as a natural estrogen compound. While our findings suggest sakuranetin contains estrogen-like properties, more investigation is required to determine the exact mechanism(s) of action and if these effects are translational. Future studies will work to compare differences in ER alpha and beta binding affinity, investigate potential SERM/SERD activity, determine bioavailability through *in vivo* studies, and identify potential elicitor-crop combinations to increase sakuranetin production.

## Supplementary Material

1

## Data Availability

Data will be made available on request.

## Appendix A. Supplementary data

Supplementary data to this article can be found online at https://doi.org/10.1016/j.jff.2026.107451.
