# Supplementary material for: Sakuranetin modulates estrogen receptor signaling in breast cancer cells
Source: J Funct Foods. Author manuscript; Available in PMC 2026 Sep 10. (PMC13556995; doi:10.1016/j.jff.2026.107451)
Supplement: Supplementary file 1 [file NIHMS2204559-supplement-1.docx]

Sakuranetin Modulates Estrogen Receptor Signaling in Breast Cancer Cells.

Jorge A. Belgodere^1,2,3^, Britney Nguyen^2^, Anna M. Hardgrove^2^, Stephen W. Wheat^1^, Isaac J. Ponder^2^, Geoffroy E.R. Sanga Pema ^1^, Julianne G. Heath^1^, William Broussard^4^, Sophie R. Dietrich^1^, Steven Elliott^1^, Syreeta L. Tilghman^5^, Muralidharan Anbalagan^3,6^, Brian G. Rowan^3,6^, Bridgette M. Collins-Burow^1,3^, Simak Ali^7^, Van H. Barnes^1,3^, Elizabeth C. Martin^1,3^, Jayalakshmi Sridhar^8^, Stephen M. Boué^9*^, Matthew E. Burow^1,3*^

^1^Tulane Department of Medicine, Section of Hematology & Medical Oncology, Tulane University Health Science Center, New Orleans, LA 70112

^2^Department of Biological and Agricultural Engineering, Louisiana State University and Agricultural Center, Baton Rouge, Louisiana 70803

^3^Tulane Cancer Center, Tulane University, New Orleans, LA 70112

^4^Oak Ridge Institute for Science and Education, U.S. Department of Energy, Oak Ridge, TN

^5^Pharmaceutical Sciences Division, College of Pharmacy and Pharmaceutical Sciences, Florida A&M University, Tallahassee, FL 32307

^6^Department of Structural and Cellular Biology, Tulane University School of Medicine, New Orleans, LA 70112

^7^Department of Surgery and Cancer, Imperial College London Hammersmith Hospital Campus London, W12 0NN, UK

^8^Department of Chemistry, Xavier University of Louisiana, New Orleans, LA 70125

^9^U. S. Department of Agriculture, Agricultural Research Service, Southern Regional Research Center, New Orleans, LA 70179

**Figure S1.** R and S molecular configurations of sakuranetin.

**Table S1.** Quantitative PCR primer sequences.

| Gene | Forward | Reverse |
| --- | --- | --- |
| β-actin | AGATGAGTATGCCTGCCGTG | ACATGTCTCGATCCCACTTAAC |
| ERα | GGCATGGTGGAGATCTTCGA | CCTCTCCCTGCAGATTCATCA |
| PGR | TACCCGCCCTATCTCAACTACC | TGCTTCATCCCCACAGATTAAACA |
| CXCL12 | ACACTCCAAACTGTGCCCTTCA | CCACGTCTTTGCCCTTTCATC |

Figure S2. Complete western blot for total PARP-1, cleaved PARP-1, and β-actin.

Figure S3. Sakuranetin elicits no estrogenic responses in cells with Y537S ER mutation. MCF-7 cells were transiently transfected with ER(2)-luc plasmid prior to treatment with DMSO, E2 (0.1 nM), ICI 182 780 (1 µM), sakuranetin (1, 10, or 25 µM). Data represents mean ± SEM for n=3 independent experiments. One-way ANOVA, Dunnett’s multiple comparisons test, compared to DMSO. ***p<0.001.
